# Mechanisms of the action of povidone-iodine against human and avian influenza A viruses: its effects on hemagglutination and sialidase activities

**DOI:** 10.1186/1743-422X-6-124

**Published:** 2009-08-13

**Authors:** Nongluk Sriwilaijaroen, Prapon Wilairat, Hiroaki Hiramatsu, Tadanobu Takahashi, Takashi Suzuki, Morihiro Ito, Yasuhiko Ito, Masato Tashiro, Yasuo Suzuki

**Affiliations:** 1 Faculty of Medicine, Thammasat University (Rangsit Campus), Pathumthani 12120, Thailand; 2 Health Science Hills, College of Life and Health Sciences, Chubu University, Kasugai, Aichi 487-8501, Japan; 3 Department of Biochemistry, Faculty of Science, Mahidol University, Bangkok, Thailand; 4 Department of Biochemistry, University of Shizuoka, School of Pharmaceutical Sciences, Shizuoka 422-8526, Japan; 5 Global COE Program for Innovation in Human Health Sciences, Shizuoka 422-8526, Japan; 6 Department of Viral Diseases and Vaccine Control, National Institute of Infectious Diseases, Toyama, Shinjuku-ku, Tokyo, 162-8640, Japan

## Abstract

**Background:**

Influenza virus infection causes significant morbidity and mortality and has marked social and economic impacts throughout the world. The influenza surface glycoproteins, hemagglutinin (HA) and neuraminidase (NA), act cooperatively to support efficient influenza A virus replication and provide the most important targets for anti-influenza chemotherapy. In this study, povidone-iodine (PVP-I), which has a broad-spectrum microbicidal property, was examined for its inhibitory effects against influenza virus infection in MDCK cells and the mechanisms of PVP-I action on HA and NA were revealed.

**Results:**

Results obtained using a novel fluorescence- and chromogenic-based plaque inhibition assay showed that 1.56 mg/ml PVP-I inhibited infections in MDCK cells of human (8 strains) and avian (5 strains) influenza A viruses, including H1N1, H3N2, H5N3 and H9N2, from 23.0–97.5%. A sialidase inhibition assay revealed that PVP-I inhibited N1, N2 and N3 neuraminidases with IC_50 _values of 9.5–212.1 μg/ml by a mixed-type inhibition mechanism. Receptor binding inhibition and hemagglutinin inhibition assays indicated that PVP-I affected viral hemagglutinin rather than host-specific sialic acid receptors.

**Conclusion:**

Mechanisms of reduction of viral growth in MDCK cells by PVP-I involve blockade of viral attachment to cellular receptors and inhibition of viral release and spread from infected cells. Therefore, PVP-I is useful to prevent infection and limit spread of human and avian influenza viruses.

## Background

Among the three types (A, B and C) of influenza viruses, A type is the most virulent, infecting various avian and mammalian species and causing human pandemics as a consequence of antigenic change (antigenic shift) in their surface glycoproteins, hemagglutinin (HA) and neuraminidase (NA) [1]. Sixteen HA and 9 NA subtypes have been recognized so far [2]. HA and NA interact with sialic acid receptors on the host cell surface, the former mediating membrane fusion that results in virus infection and the latter possessing sialidase activity that cleaves sialyl linkages between viral HA and cellular receptors to release progeny viruses and separate viruses from HA-mediated self-aggregation, allowing the virus to infect a new host cell for continuing virus replication [3].

Virus infection can be inhibited by the use of compounds that bind to viral HA [4-6], inhibit NA activity [7-11] or inhibit both HA and NA activities [12]. Two NA inhibitors, sialic acid and shikimic acid analogues, have recently been licensed for treatment of influenza A and B infections: zanamivir [13] (Relenza^®^), which is administered by inhalation, and oseltamivir phosphate [14] (Tamiflu^®^), which is administered orally as a prodrug and is converted by hepatic esterase to its active form, oseltamivir carboxylate (OC). However, influenza A and B viruses with mutations in the NA gene have developed resistance to oseltamivir and zanamivir [15,16]. The worldwide circulation of oseltamivir-resistant seasonal H1N1, highly pathogenic avian H5N1 [17,18] and the pandemic (H1N1) 2009 [19] have provided an impetus to develop new antiviral and antiseptic materials.

In the nineteenth century, povidone-iodine (PVP-I), a polyvinylpyrrolidone iodine complex, was developed and found to have a potent broad-spectrum activity against bacteria, mycobacteria, fungi, viruses and protozoa [20]. PVP-I has become widely used as an antiseptic and disinfectant. Despite long-term use, development of PVP-I resistance in microorganisms has not been reported [21,22].

PVP-I products have been found to be effective in inactivating a variety of enveloped and nonenveloped viruses, such as polio [23], herpes simplex, herpes zoster [24], and human immunodeficiency viruses [25,26]. Anti-influenza virus activity of PVP-I also has been reported recently [26-28]. Pretreatment of avian influenza H5N1, H5N3, H7N7 and H9N2 viruses with PVP-I products, such as solution, scrub, gargle and throat spray, in the range of 0.23–2%, reduced viral infectious titers to undetectable values in embryonated hen's eggs [27]. Both aqueous (Betaisodona^®^) and liposomal PVP-I inactivated human influenza A virus (H3N2), resulting in reduction of the virus titer by more than 4 orders of magnitude in Madin-Darby canine kidney (MDCK) cells [28]. However, the target sites and mechanisms of PVP-I action on influenza A and the other virus infections have hitherto remained unknown. In this study, we investigated mechanisms underlying PVP-I anti-influenza activity. The apparent reduction of influenza A viral infectious titers after incubation with PVP-I products within a short period of time [26-28] led us to investigate two spike glycoproteins on the viral surface, HA and NA, which play essential roles in viral infection, as targets of PVP-I anti-influenza effects.

## Results

### Inhibition by PVP-I of influenza A virus growth in MDCK cells

**Figure 1:**
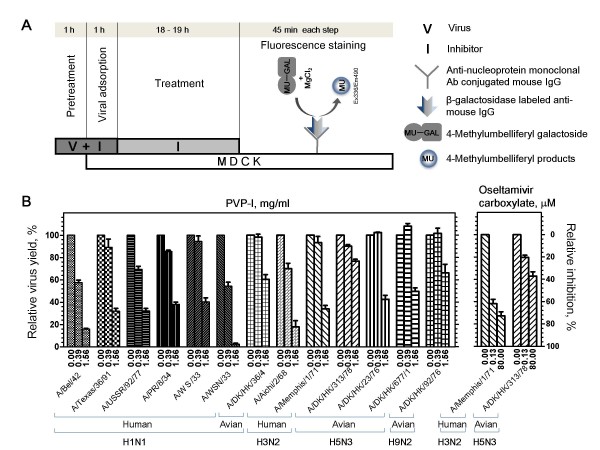
**Inhibitory effect of PVP-I on influenza viral infection in MDCK cells**. (A) A simplified diagram of the infection assay used in this study. (B) Quantification of viruses in cells at Ex355/Em460 is expressed as percent virus yield (left Y axis) and percent inhibition (right Y axis) of untreated infected cells.

We first determined the cytotoxicity of PVP-I against MDCK cells employed as host cells of influenza viruses in this study by using a cell counting kit-8 assay. Half-maximum cytotoxic concentration of PVP-I after 24-h exposure of MDCK cells to PVP-I was 2.4 ± 0.2 mg/ml. PVP-I ranging from 0–1.56 mg/ml, which had no effect on MDCK cells, reduced virus yield in MDCK cells in a dose-dependent manner (Figure 1B). In comparison with virus yield in the absence of the inhibitor, 1.56 mg/ml of PVP-I reduced human virus yield by 59.7–97.5% and avian virus yield by 23.0–57.4%, suggesting enhanced sensitivity towards human viruses compared to that toward avian viruses. OC, used as control, inhibited A/Memphis/1/71 (H3N2) infection by 62% and 73% at concentrations of 0.13 μM and 80 μM, respectively, whereas it inhibited A/DK/HK/313/78 (H5N3) infection by 20% and 37%, respectively, at the same concentrations.

### Binding of influenza A viruses to sialoglycopolymers and guinea pig erythrocytes and inhibition by PVP-I

**Figure 2:**
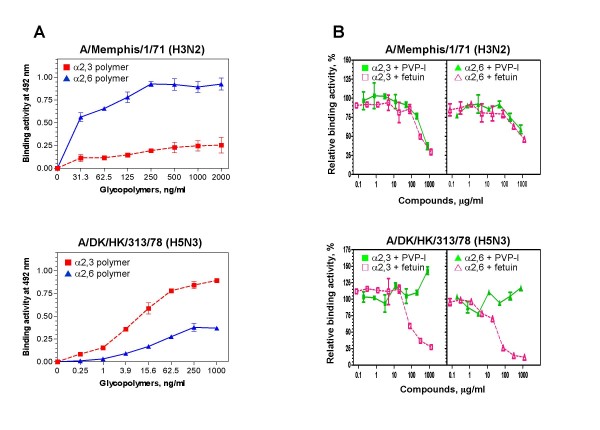
**Effect of PVP-I on direct binding activity of influenza viruses to glycopolymers**. (A) Virus binding activity to glycopolymers linked with α2,3 (filled red square) and α2,6 (filled blue triangle)-sialic acids. (B) Inhibition of virus binding to a specific polymer. Percentage of untreated control viruses was plotted against inhibitor concentration. (filled green square) α2,3 linkage + PVP-I; (empty pink square) α2,3 linkage + fetuin; (filled green triangle) α2,6 linkage + PVP-I; (empty pink triangle) α2,6 linkage + fetuin.

In agreement with hemagglutinins from avian and human influenza viruses, which prefer binding to α2,3- and α2,6-sialylated polymers, respectively [29], A/Memphis/1/71 and A/DK/HK/313/78 viruses predominately bound to sialoglycopolymers terminated in α2,6 and α2,3 respectively (Figure 2A). Binding of A/Memphis/1/71 to α2,3 and α2,6 polymers was reduced by fetuin (up to 1.25 mg/ml) and PVP-I (up to 0.78 mg/ml), whereas that of A/DK/HK/313/78 was inhibited by fetuin but not by PVP-I (Figure 2B).

**Figure 3:**
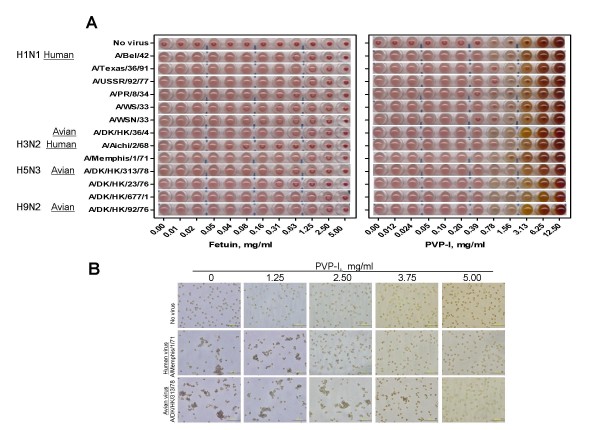
**Inhibition by PVP-I of influenza virus hemadsorption activity on guinea pig erythrocytes**. (A) Quantification of minimum fetuin (left panel) or PVP-I (right panel) concentration required for inhibition of virus erythrocyte agglutination. (B) Visualization of virus erythrocyte agglutination under a light microscope. Bars = 50 μm. Agglutination morphology was compared with positive (no PVP-I) and negative (no virus) controls.

Quantitative inhibition of viral HA binding to sialo-glycoconjugate receptors on the erythrocyte surface by fetuin control and PVP-I is shown in Figure 3A and summarized for PVP-I activity in Table 1. No erythrocyte hemolysis and no significant change in pH (pH of each well ranging from 6.52 to 7.20) in the assay system were observed. In general, fetuin exhibited higher inhibitory activity (ranging from 0.02 to 1.25 mg/ml) than that of PVP-I (0.2–12.5 mg/ml).

**Table 1:** Inhibition by PVP-I of sialidase activity, hemagglutination and infectivity activity of influenza A viruses

**Virus subtype**	**Virus strain**	**Sialidase inhibition activity** **IC_50_^a ^(μg/ml)**	**Hemagglutination inhibition activity^b ^(mg/ml)**	**Infection inhibitory activity (%)^c^**
H1N1	A/Bel/42	11 ± 2	1.56	84 ± 1
	A/Texas/36/91	72 ± 4	0.78	68 ± 3
	A/USSR/92/77	47 ± 3	0.78	68 ± 2
	A/PR/8/34	9.5 ± 0.5	0.20	62 ± 2
	A/WS/33	12.5 ± 0.5	0.78	60 ± 4
	A/WSN/33	45 ± 1	0.39	97 ± 1
	
	A/DK/HK/36/4	21 ± 5	12.50	40 ± 4

H3N2	A/Aichi/2/68	96 ± 2	3.13	82 ± 5
	A/Memphis/1/71	61 ± 4	1.56	66 ± 3

H9N2	A/DK/HK/92/76	212 ± 9	12.50	34 ± 8

H5N3	A/DK/HK/313/78	78 ± 4	12.50	23 ± 1
	A/DK/HK/23/76	124 ± 7	3.13	57 ± 4
	A/DK/HK/677/1	55 ± 1	3.13	50 ± 3

^a^IC_50_values are concentrations inhibiting viral sialidase activity by 50%. Standard error means were calculated from means of two independent experiments, each conducted in duplicate.

^b^Minimum concentration that inhibits hemagglutination.

^c^Percent viral inhibition (with 1.56 mg/ml of PVP-I) was calculated by comparison with the control without an inhibitor. Each experiment was performed in triplicate.

A qualitative analysis of hemagglutination inhibition showed that hemagglutination (guinea pig erythrocyte clumping) of human A/Memphis/1/71 (~400 hemagglutination units (HAU)) and avian A/DK/HK/313/78 (~400 HAU) was completely inhibited by 2.50 mg/ml and 5.00 mg/ml of PVP-I, respectively (Figure 3B).

### Effect of PVP-I on influenza A virus sialidase activity

In order to examine the effect of PVP-I on sialidase activity of different subtypes of influenza virus strains, the enzyme activity and K_m _value of each virus subtype were determined at pH 6.0 using 2'-(4-methylumbelliferyl)-α-D-*N*-acetylneuraminic acid (MUNA), a sensitive fluorogenic substrate without 2,3 and 2,6 linkages. Then an inhibition assay was performed using 2 enzyme units of each virus subtype and substrate concentration at its K_m _value. IC_50 _values of OC against sialidase of different virus strains were ranged from 0.37 to 6.88 nM (data not shown). There were marked differences in IC_50 _values for PVP-I, from 9.5 to 212.1 μg/ml depending on the virus strain (Table 1).

**Figure 4:**
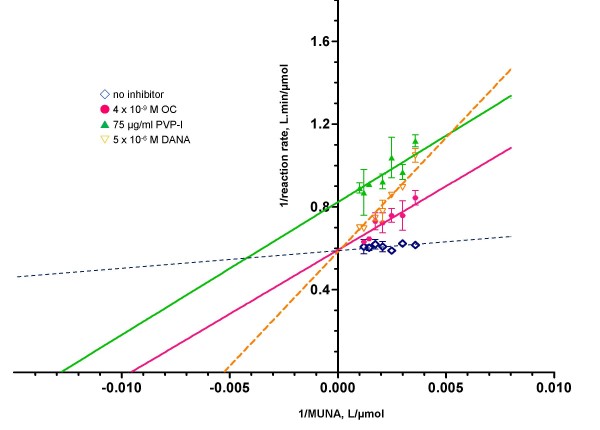
**Lineweaver-Burk plots of inhibition of A/PR/8/34 (H1N1) sialidase activity by OC, DANA and PVP-I**. (empty blue diamond) no inhibitor; (filled pink circle) 4 × 10^-9 ^M OC; (filled green triangle) 75 μg/ml PVP-I; (empty orange upside down triangle) 5 × 10^-6 ^M DANA.

The kinetic mechanism by which PVP-I inhibits influenza A virus sialidase activity was investigated by determining kinetic parameters of human A/PR/8/34 (H1N1) sialidase on hydrolysis of MUNA in the absence and presence of an inhibitor. As shown in Table 2, with OC or 2-deoxy-2,3-dehydro-*N*-acetylneuraminic acid (DANA), K_m _values increased, but V_max _did not change. In the presence of PVP-I, K_m _values increased and V_max _decreased. V_max_/K_m _ratio decreased 6-fold, 6-fold and 12-fold in the presence of 4 nM OC, 75 μg/ml PVP-I and 5 μM DANA, respectively, indicating decrease in sialidase efficiency. Lineweaver-Burk plots showed that inhibition of A/PR/8/34 sialidase activity by OC and DANA was of a competitive type, whereas that by PVP-I was of a mixed type (Figure 4). The K_i _values for free sialidase for OC, DANA and PVP-I were 0.66 nM, 432.60 nM and 11.74 μg/ml, respectively, and the K_i _for sialidase-MUNA complex for PVP-I was 190.63 μg/ml, whereas K_m _for MUNA was 14.66 μM (7.17 μg/ml). Thus, the competitive inhibitors OC and DANA exhibited 2.21 × 10^4^- and 34-fold higher affinities for influenza sialidase, respectively, than that of MUNA, whereas the mixed-type inhibitor PVP-I, with two inhibition constants, K_i _for free sialidase and K_is _for bound sialidase complex, had 1.6- and 26.6-fold lower affinities than that of MUNA, respectively.

**Table 2:** Effects of inhibitors on kinetics parameters of A/PR/8/34 (H1N1) sialidase activity

**Kinetic parameter**	**No inhibitor**	**OC** **(4 nM)**	**PVP-I** **(75 μg/ml)**	**DANA** **(5 μM)**
V_max_(μmol/l.min)	1.70	1.68	1.22	1.69
K_m _(μmol/l)	14.66	103.20	77.72	184.10
K_m _(μg/ml)^a^	7.17	-	-	-
V_max_/K_m_(1/min)	0.12	0.02	0.02	0.01

Type of inhibition	-	Competitive	Mixed	Competitive

K_i_(nM)	-	0.66	-	432.60
K_i _(μg/ml)	-	-	11.74	-
K_is _(μg/ml)	-	-	190.63	-

K_m_/K_i_	-	2.21 × 10^4^	0.61	33.90
K_m_/K_is_	-	-	0.04	-

^a^MW of MUNA is 489.39.

## Discussion

Iodine is a nonmetallic essential nutrient with a potent broad range of microbicide actions against almost all of the important health-related microorganisms, including bacteria, fungi, viruses and protozoa. Although a high content of iodine species with free molecular form (I_2_) and hypoiodous acid (HOI) in aqueous solution has powerful microbicidal effects but can cause volatility, stinging and cytotoxicity [30-32]. To overcome these problems, iodine was combined with neutral carrier polymers to increase iodine solubility and to keep low the release of iodine as a solubilizing agent and to act as an iodine reservoir [30,33]. The most popular carrier in current use is povidone [32,33], which has no microbicidal activity [34]. Since povidone slowly and continuously releases free iodine into solution, these properties help to maintain antimicrobial capacity for a long period and to decrease toxicity.

By using the cell counting kit-8 assay, we found that the IC_50 _cytotoxicity of MDCK cells following 24-h exposure to PVP-I was 2.4 ± 0.2 mg/ml. Based on morphological criteria [35], cell shrinkage, rounding and detachment from the surface of the culture plate after treatment with 3.1 mg/ml of PVP-I suggested that the cells were undergoing apoptosis. Therefore, we used low concentrations of PVP-I that did not cause any toxicity to host MDCK cells in order to investigate its anti-influenza virus activity.

Our results confirmed that PVP-I is a potent inhibitor of influenza virus production in MDCK cells. We indicated that PVP-I inhibits the viral replication in a dose dependent manner and is more active against human viruses (H1N1, H3N2) than avian viruses (H1N1, H5N3, H9N2). PVP-I appeared to inhibit binding of human A/Memphis/1/71 (H3N2) virus to specific sialoglycopolymers but not that of avian A/DK/HK/313/78 (H5N3) virus. Hemagglutination of erythrocytes induced by human viruses was inhibited by PVP-I, while hemagglutination inhibition of avian viruses required higher PVP-I concentrations. Differences in hemagglutination inhibitory activity of PVP-I against various viruses may be associated with the different structure of HA protein of each virus type. Unlike the α2,3 and α2,6 sialoconjugated protein fetuin [36], which reduces HA binding activity of both avian and human influenza viruses via competition for binding with sialyloligosaccharide receptor substrates to the viruses [37], blockage of viral HA attachment to receptor substrates by PVP-I may result from alteration of viral HA protein structure by reaction of free iodine with basic -NH groups, phenolic groups, and -SH groups of amino acid residues [30]. Although avian viruses appear to be less sensitive than human viruses to PVP-I, based on results of the erythrocyte agglutination assay, which reflects viral attachment to host cells, agglutination of avian A/DK/HK/313/78 virus (~400 HAU) was completely inhibited after a second exposure to 5 mg/ml of PVP-I. This is in agreement with the finding that titers of a highly pathogenic avian virus (H5N1) and three low pathogenic avian viruses (H5N3, H7N7 and H9N2) cultivated in embryonated eggs become undetectable by incubation with a commercial PVP-I product for 10 seconds before inoculation [27]. These results suggest that gargling with PVP-I could prevent human infection not only by human influenza viruses that bind to sialyl α2,6 Gal receptors in the upper part of human trachea but also by avian viruses that bind to sialyl α2,3 Gal receptors that exist deep in the human respiratory tract [38]. This could consequently minimize the risk of avian virus mutation, either by adaptation or reassortment, to recognize the human host predominately carrying α2,6-linked sialic acids.

PVP-I inhibited sialidase activity as a mixed-type inhibitor, indicating that free iodine is capable of binding to either free sialidase or sialidase complexed with its substrate, but iodine binding to free sialidase is more efficient than that to sialidase-substrate complex as K_i _was 16-fold lower than K_is_. This may be explained by the distribution of lysine, arginine, histidine, cysteine and tyrosine residues throughout the sequence of the NA molecule, which are reactive with iodine [30]. Although the K_i _value for iodine was higher than the K_m _value, indicating that affinity of the MUNA substrate for sialidase is higher than iodine, the activity of sialidase to hydrolyze MUNA (V_max_/K_m _= 0.12) was reduced in the presence of PVP-I (75 μg/ml) (V_max_/K_m _= 0.02), comparable to that in the presence of OC (4 nM). The reduction in sialidase activity should result in a decrease in influenza replication.

There have been a number studies on the development of harmless carriers (such as cyclodextrin) that slowly release free iodine at a concentration retaining antimicrobial activity without a cytotoxic effect against mammalian cells for use of iodine in therapeutic applications [28,39]. Intravenous administration of iodine-lithium-α-dextrin has successfully prevented lethal infection of *Staphylococcus aureus *in rats [39].

## Conclusion

Our study confirms the inhibition of avian and human influenza A virus infection by PVP-I and demonstrates that PVP-I inhibits both viral HA binding activity and viral NA catalytic hydrolysis, mediating virus entry into host cells, and virion release and spread to a new host cell, respectively. Thus, PVP-I, for which there has been no report of resistance, is a potential agent that not only prevents viral infections but also reduces the spread of influenza viruses in epidemic and pandemic areas.

## Methods

### Viruses and cells

Viruses were propagated in 10-day-old embryonated chicken eggs at 34°C, and after 48 h of incubation, allantoic fluid was harvested, cleared and concentrated. The virus pellet was resuspended in cold phosphate-buffered saline (PBS), divided into aliquots, and kept at -80°C until use. Virus titers expressed as HAU were determined (see below) before experimentation. Protein concentration was determined by using a BCA™ protein assay kit (Pierce, Rockford, IL, USA) with bovine serum albumin (BSA) as a standard. MDCK cells were cultured in Eagle's minimal essential medium (EMEM) supplemented with 5% fetal calf serum, antibiotics (penicillin-streptomycin) and glutamine at 37°C in an atmosphere of 5% CO_2_.

### Monoclonal antibodies

Monoclonal antibody 4E6 (mouse IgG1 subtype) directed to influenza virus nucleoprotein (NP) was obtained using A/Memphis/1/71 (H3N2) as an antigen. Monoclonal antibody 2E10 (mouse IgG1 subtype) directed to H3 HA and 2G3 (IgG1) directed to H5 HA was prepared using reassortant virus A/Memphis/1/71 (H3)-A/Bellamy/42 (N1) and A/duck/HK/313/4/78 (H5N3) as an antigen, respectively [40].

### Cytotoxicity assay (Cell counting kit-8 assay)

Toxicity of PVP-I (Meiji Seika Kaisha, Tokyo, Japan) against MDCK cells was examined using a cell counting kit-8 (DOJINDO Laboratories, Kumamoto, Japan) to determine the numbers of viable cells using WST-8 [2-(2-methoxy-4-nitrophenyl)-3-(4-nitrophenyl)-5-(2,4-disulfophenyl)-2H-tetrazolium, monosodium salt] as a substrate. In brief, confluent monolayers of MDCK cells in a 96-well plate were washed with serum-free EMEM and incubated with PVP-I at a final concentration ranging from 0–25 mg/ml for 24 h at 37°C in 5% CO_2 _atmosphere. After removal of PVP-I solution, cell viability was determined by addition of kit reagent, incubation for 1 h at 37°C, and measurement of absorbance at 450 nm. PVP-I concentrations showing no cytotoxicity against MDCK cells were used in viral infection assay.

### Viral infection inhibition assay

Inhibitory effects on virus development in MDCK cells were determined as shown in Figure 1A. An aliquot of virus (V) at the optimal dose (input virus causing 5 × 10^5 ^fluorescent forming units, which corresponds to 250 PFU/well equivalent to a multiplicity of infection (MOI) of 0.025 PFU/cell, in the absence of an inhibitor [41]) was preincubated with an equal volume of serial dilutions of an inhibitor (I) or serum-free EMEM (100% infection) at 4°C for 1 h (Pretreatment step). A 100-μl aliquot of virus-inhibitor mixture (V+I) was overlaid on MDCK cells in 96-well plates at 34°C for 1 h (Viral adsorption step). After washing with serum-free EMEM, virus-infected cells were cultured in serum-free EMEM containing 2.5 μg/ml acetylated trypsin with or without the inhibitor (at the same concentration as that in preincubation) for 18–19 h at 34°C (Treatment step). Then the cell monolayer was washed with PBS, fixed with methanol for 1 min, and incubated with anti-nucleoprotein mouse IgG (4E6) for 45 min at room temperature. β-galactosidase-labeled anti-mouse IgG with 0.1% block-ace was added and incubated for 45 min at room temperature. Then the enzyme reaction was started by adding 40 μM 4-methylumbelliferyl-β-D-galactoside (MU-Gal) with 50 μM MgCl_2_. After 45 min of incubation at 37°C, the reaction was stopped by addition of 100 mM sodium carbonate buffer (pH 10.6). Fluorescent intensity of released 4-methylumbelliferone (MU) was measured by excitation at 336 nm and emission at 490 nm (Ex355/Em460) using a fluorescence microplate reader (Fluorescence staining step).

### Hemagglutination and hemagglutination inhibition assays

For virus quantitation, a 5-μl aliquot of virus sample serially diluted in PBS was incubated with 50 μl of 0.5% suspension of guinea pig erythrocytes for 2 h at 4°C. Virus titers expressed as HAU were determined by visual reading.

For determination of inhibition of HA binding, a 25-μl aliquot of fetuin or PVP-I serially diluted in PBS was preincubated with 25 μl of virus (4 HAU) in 96-well U-bottom plates at 4°C for 1 h. Hemagglutination was determined as described above.

To visualize agglutination directly, 100 mg/ml of PVP-I was added to a well of a 24-well flat-bottom plate to give a final concentration of 1.25, 2.50, 3.75 or 5.00 mg/ml, followed by addition of 5 μl of virus (2^14 ^HAU) to obtain a final HAU of about 400 [42]. To avoid possible cleavage of virus-erythrocyte binding by sialidase, erythrocyte agglutination was observed under a light microscope immediately after addition of 200 μl of 0.25% erythrocyte suspension.

### Receptor binding inhibition assay

Specific binding of virus to α2,3 and α2,6 sialoglycopolymers was performed in 96-well plates coated with 0–2,000 ng/ml of sialoglycopolymers as described previously [43]. Briefly, after blocking the plates with 1% block-ace in PBS, viruses (64 HAU) in PBS containing 0.1% Tween 20 (PBST) were added and incubated for 2 h at 4°C. Viruses bound to sialoglycopolymers were detected by anti-HA mouse IgG and HRP-conjugated goat anti-mouse IgG+M, each step being conducted at 4°C for 2 h and followed by washing with PBST 5 times. The reaction color was developed by adding *o*-phenylenediamine (OPD) and H_2_O_2 _in 100 mM citrate buffer (pH 6.0), prepared according to the instruction manual (Wako, Osaka, Japan), terminated by adding H_2_SO_4_, and assessed by measuring optical densities at 492 and 620 nm.

A receptor inhibition experiment was performed as described above, but each virus (12 HAU) was preincubated with PVP-I (0–780 μg/ml) or fetuin (0–1,250 μg/ml) in PBST at 4°C for 2 h before the virus mixture was added to the plate coated with 50 ng/ml of sialoglycopolymer.

### Sialidase inhibition assay

Five μl of 2 viral enzyme units (one unit being the amount required to liberate 1 nmole of product per min) was preincubated with 5 μl of an inhibitor serially diluted in 20 mM sodium acetate buffer (pH 6.0) at 37°C for 15 min. Enzymatic reaction was started by addition of 5 μl of MUNA to a final concentration equivalent to a K_m _value of each virus. After 15 min at 37°C, the reaction was terminated by adding 200 μl of 100 mM sodium carbonate buffer (pH 10.6). The released MU products were measured at Ex355/Em460.

To determine the inhibition mechanism, 5 μl of 2 viral enzyme units was incubated with 5 μl of 280–1000 μM MUNA either alone or in the presence of 5 μl of OC, DANA or PVP-I for 15 min at 37°C. The MU products were measured as described above. Data were fitted to the Michaelis-Menten equation for determination of apparent Vmax and K_m _values and then transformed and plotted as Lineweaver-Burk plots using GraphPad Prism software. Inhibition constant of each inhibitor was calculated according to their inhibition type as follows:

For competitive inhibitor, K_mapperance _= K_m_(1+I/K_i_).

For mixed-type inhibitor, V_maxapperance _= V_max_/(1+(I/K_is_)) and K_mapperance _= K_m_(1+I/K_i_)/(1+I/K_is_).

K_i _and K_is _are dissociation constants for free enzyme-inhibitor and substrate-bound enzyme-inhibitor complex, respectively.

## Competing interests

The authors declare that they have no competing interests.

## Authors' contributions

Conceived and designed the experiments: NS, YS, PW, MT. Performed the experiments: NS, TT. Analyzed the data: NS, YS, TT, TS. Contributed reagents/materials/analysis tools: YS, MT, HH, MI, YI. Wrote the paper: NS, YS, PW. All authors have read and approved the final manuscript.
